# The present and future of seizure detection, prediction, and forecasting with machine learning, including the future impact on clinical trials

**DOI:** 10.3389/fneur.2024.1425490

**Published:** 2024-07-11

**Authors:** Wesley T. Kerr, Katherine N. McFarlane, Gabriela Figueiredo Pucci

**Affiliations:** Department of Neurology, University of Pittsburgh, Pittsburgh, PA, United States

**Keywords:** epilepsy, wearables, internet of things, human-in-the loop, deficiency time

## Abstract

Seizures have a profound impact on quality of life and mortality, in part because they can be challenging both to detect and forecast. Seizure detection relies upon accurately differentiating transient neurological symptoms caused by abnormal epileptiform activity from similar symptoms with different causes. Seizure forecasting aims to identify when a person has a high or low likelihood of seizure, which is related to seizure prediction. Machine learning and artificial intelligence are data-driven techniques integrated with neurodiagnostic monitoring technologies that attempt to accomplish both of those tasks. In this narrative review, we describe both the existing software and hardware approaches for seizure detection and forecasting, as well as the concepts for how to evaluate the performance of new technologies for future application in clinical practice. These technologies include long-term monitoring both with and without electroencephalography (EEG) that report very high sensitivity as well as reduced false positive detections. In addition, we describe the implications of seizure detection and forecasting upon the evaluation of novel treatments for seizures within clinical trials. Based on these existing data, long-term seizure detection and forecasting with machine learning and artificial intelligence could fundamentally change the clinical care of people with seizures, but there are multiple validation steps necessary to rigorously demonstrate their benefits and costs, relative to the current standard.

## Highlights

- Seizure detection, prediction, and forecasting technologies can be evaluated based on sensitivity, false positive rate, and deficiency time.
- Performance of these technologies must be evaluated on unseen data.
- Seizure detection technologies can have high sensitivity for motor seizures but less for other seizure types.
- Seizure prediction and forecasting can substantially improve quality of life in people with epilepsy.

## 1 Introduction

A seizure is defined by the Oxford dictionary as a sudden attack of illness (1). The sudden nature of symptoms is a key component of the disability incurred, as measured both by reduced quality of life and increased mortality rates (2–4). For people with transient neurological events, the first question is if the event represents an epileptic seizure, functional (nonepileptic) seizure, or non-epileptic non-functional event (e.g., convulsive syncope) (5, 6). The differentiation of these transient neurological events can be challenging without simultaneous video-electroencephalographic monitoring (VEM): 30% of patients with presumed epilepsy who undergo VEM instead have functional seizures and 10% of patients who present for prolonged seizure to emergency rooms in clinical trials had functional and not epileptic seizures (7–9). VEM often requires hospitalization, tends to last for less than 10 days at a time, and is primarily available at tertiary care centers (10). Therefore, one key clinical challenge is to develop hardware and software technologies for highly accurate, reliable, and long-term detection of epileptic seizures.

In addition to differentiation of epileptic seizures from non-epileptic transient neurological events, there are substantial challenges in counting epileptic seizures in people with known epilepsy. Obtaining an accurate and reliable count of epileptic seizures is a foundational aspect of making treatment decisions for people with epilepsy because people with continued epileptic seizures may require alteration of treatment to reduce or eliminate epileptic seizures. The seizure counts provided by patients, witnesses, or care partners have been the basis for clinical decision making and rigorous evaluation of antiseizure medications (ASMs) and non-pharmaceutical treatments for decades. However, human-provided seizure counts have limited sensitivity, especially for focal unaware seizures where the lapse in formation of new memories during the seizure also means that the patient can forget the seizure itself (11, 12). In addition, retrospective seizure counts may represent the overall gestalt of the patient and care partner regarding the effectiveness of treatment. Using the principles of the placebo effect, people may underestimate seizure counts when there is confidence in the treatment (13–19). Conversely, skepticism or adverse effects may contribute to nocebo effect that overestimates seizure counts (18, 20–22). In long-term clinical practice, simulation-based studies suggest that the long-term dose and number of ASMs were not substantially impacted by reduced sensitivity and false positive rate, as long as sensitivity was at least 10% (23). In short term clinical trials that aim to determine treatment response within weeks to months, statistical power could substantially improve with seizure counts based on intracranial electroencephalographic (EEG) devices, but the impact of less invasive devices may be less (23–25). Conversely to patients with continued seizures, people who are seizure free may either continue current treatment, reduce the intensity or dose of current treatments, or withdraw treatment. While there are numerous barriers to withdrawal of ASMs in people who are seizure free, this uncertainty in counting seizures may contribute to the remarkably low rate of ASM withdrawal in people with long-term seizure freedom (26).

In addition to seizure detection, if a person with epilepsy was able to reliably predict when a seizure would occur, then they could take safety precautions (e.g., pull over an automobile) or tailor their treatment based on this risk (e.g., take a rescue medication or receive responsive neurostimulation) (27–29). The severe and unpredictable nature is one challenge in addressing the fact that people with epilepsy have a 3-to-12-fold increased risk of death for all causes, as well as Sudden Unexpected Death in Epilepsy (SUDEP) (2). Currently, the clinical care of patients with seizures focuses on reducing this risk by achieving seizure freedom.

One goal of the treatment of seizures is to allow the person with seizures to live their life as if they did not have seizures by the treatment causing both seizure freedom and no adverse effects. However, in more than one third of people with seizures, antiseizure medications fail to achieve seizure freedom (30); therefore, clinical care focuses on maximizing quality of life by reducing seizure frequency, minimizing adverse effects of medications, and addressing comorbidities or complications of seizures (4, 31, 32). In people with medication-resistant epilepsy, this quality of life could be massively improved if they could reliably predict when a seizure would not occur so they can engage in valuable and rewarding activities that otherwise would not be safe (e.g., driving and swimming) (33, 34).

It is both challenging and not useful to make seemingly definite binary (yes/no) predictions of if someone will have a seizure (33, 34). Therefore, instead of seizure prediction, the approach changed to provide individualized seizure forecasts instead of predictions (35–39). Similar to forecasting the weather, seizure forecasts aim to reliably identify these high and low risk states, while recognizing that their predictions likely are imperfect. If a person has one seizure a year in the low-risk state and one seizure a day in the high-risk state, then they could take meaningful precautions based on this forecast.

To develop these forecasts, clinicians and researchers have developed long-term monitoring hardware and software to both differentiate seizures from mimics as well as identify pre-seizure states (35, 37, 40). The hardware aims to measure signals from the person with seizures over time that are consistent, reliable, and have minimal noise so that the software can use data-driven techniques like machine learning and artificial intelligence (41, 42). Machine learning (ML) tools are trained based on historical data to maximize their performance based on a single quantitative metric (e.g., accuracy of classifying seizure [ictal], pre-seizure [pre-ictal], post-seizure [post-ictal], versus between seizure [interictal] states). Artificial intelligence (AI) tools are designed to perform a broad range of tasks, including tasks for which they have not been explicitly trained, and can do so using multiple ML tools (43). We are not aware of any AI tool for seizures that both fills that definition and is approved or cleared for clinical use by the United States Food and Drug Administration (FDA). There are numerous multiple FDA-cleared ML algorithms applied to data relevant to seizures and ML-based devices (see Sections 3–5).

While there is substantial hype about the power of ML/AI for clinical medicine (44, 45), especially seizures, there are only a limited number of tools that have rigorously demonstrated their utility and have been integrated into standard clinical practice (43). This is despite decades of effort, especially focused on seizure detection and prediction using electroencephalography (EEG) (46, 47). In order to understand the future of ML/AI for clinical practice, we must then understand the benefits and limitations of the existing approaches.

The central focus of this narrative review is highlighting the existing software and hardware for the detection and forecasting of seizures. Before discussing that existing software and hardware, we provide context by discussing key aspects of how these technologies’ performances should be evaluated by a clinician, person with seizures, or patient-advocate. Afterwards, we discuss the implications for these technologies on the design and conduct of clinical trials for treatments of seizures.

## 2 How to evaluate new machine learning tools for seizure detection, prediction, and forecasting

Ideally, seizures are rare events. Therefore, while there are commonalities between seizures and other applications of ML/AI, there are additional unique considerations (48).

### 2.1 Training, testing, and validation sets

**Figure 1:**
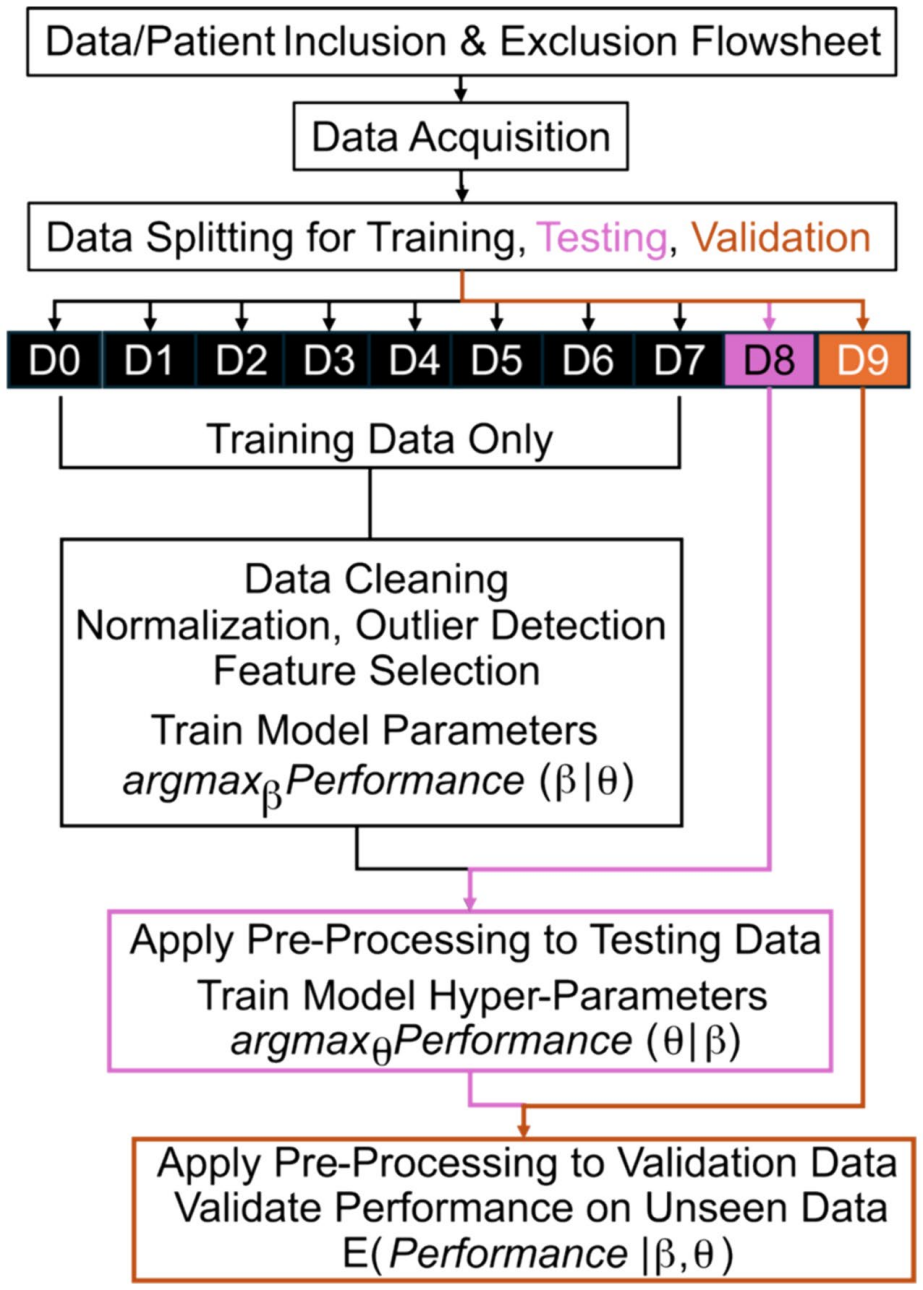
Machine learning training, testing, and validation flowsheet. The best parameters, *β*, of a model that maximize a chosen quantitative metric of performance are learned based on training from training data only. After application of all pre-processing steps to the testing data without modification, the best hyperparameters, *θ*, of a model that maximize performance are learned based on testing data, without modification of the learned parameters, *β*. Lastly, the expected (E) performance is measured based on validation data after application of all pre-processing steps and applying the model with the optimized parameters, *β*, and hyperparameters, *θ*. D# reflects a numbered subset of data; argmax reflects identifying the optimal argument (arg) that maximizes (max) the performance; the vertical line, |, means “given” in mathematical notation.

Fundamentally, ML/AI are data-driven techniques to understand patterns in historical data that are then applied to unseen data (48). When these tools are being developed, their designers should be explicit regarding what data are used for training, testing, and validation (Figure 1). We define training data as data used to learn parameters of the model (e.g., odds ratios within logistic regression). While not necessary for all applications, testing data are used to learn hyperparameters or higher-level structures within the model that are not effectively optimized simultaneous with parameters (e.g., which ML model to use). Lastly, validation data are used to evaluate the performance of the model on “unseen” data and, thereby, validation data should never overlap with training or testing data.

A common error in the development of ML/AI is “leakage” or “peeking” where validation data leaks into the process of training or testing. For example, consider an ML tool that detects seizures based on accelerometry from a wearable device on the wrist, similar to a watch (49, 50). If developers report the performance of two ML approaches, support vector machines (51) and a neural network (52), on the “validation” data, then they can perform statistical tests to determine if one ML approach achieved superior performance. However, this “validation” data could be better described as “testing” data because they do not separately apply the better algorithm to “unseen” data. The performance on the “testing” data can be inflated as compared to truly unseen data because the developers tested two approaches and chose the better approach by “peeking” at the performance on validation data, which incurs a similar bias to multiple testing in null-hypothesis significance testing (53).

While that version of “peeking” is now less prevalent than before, a common error observed in peer review of these algorithms is “leakage” during pre-processing and feature selection (53). ML/AI tools can be described as data-hungry because their ability to make reliable predictions is non-linearly and highly dependent on the size and diversity of the training data (54). When developers have access to long-term recordings, they often can derive hundreds, thousands, or even millions of quantitative metrics, *p*, that could predict if an event was a seizure. Because seizures are rare, it can be more challenging to have a similarly large number of examples of seizure and not seizure, *n*. To train stable ML/AI models, many statistical techniques require that *p* be less than *n* and, ideally, *p* is much less than *n*. To address this, developers use their biological and technical knowledge to select features that may be reliably measured and related to seizures (e.g., placing a wearable on the arm that shakes during a motor seizure) (55), in combination with statistical techniques to find the features with the best performance (56). One simple technique can include mass univariate null-hypothesis testing, where each of the *p* candidate features is compared in examples of seizures as compared to not seizures (57). The subsequent ML/AI tool could erroneously base its final prediction on the combination of these top features (57). This is an error because the “validation” data “leaks” into training data: validation data is used to rank the *p* candidate features! This “leakage” also inflates the performance of the ML/AI tool in unpredictable ways (53); therefore, developers should be extremely explicit to clarify which data contributed to each stage of training, testing, and validation.

**Figure 2:**
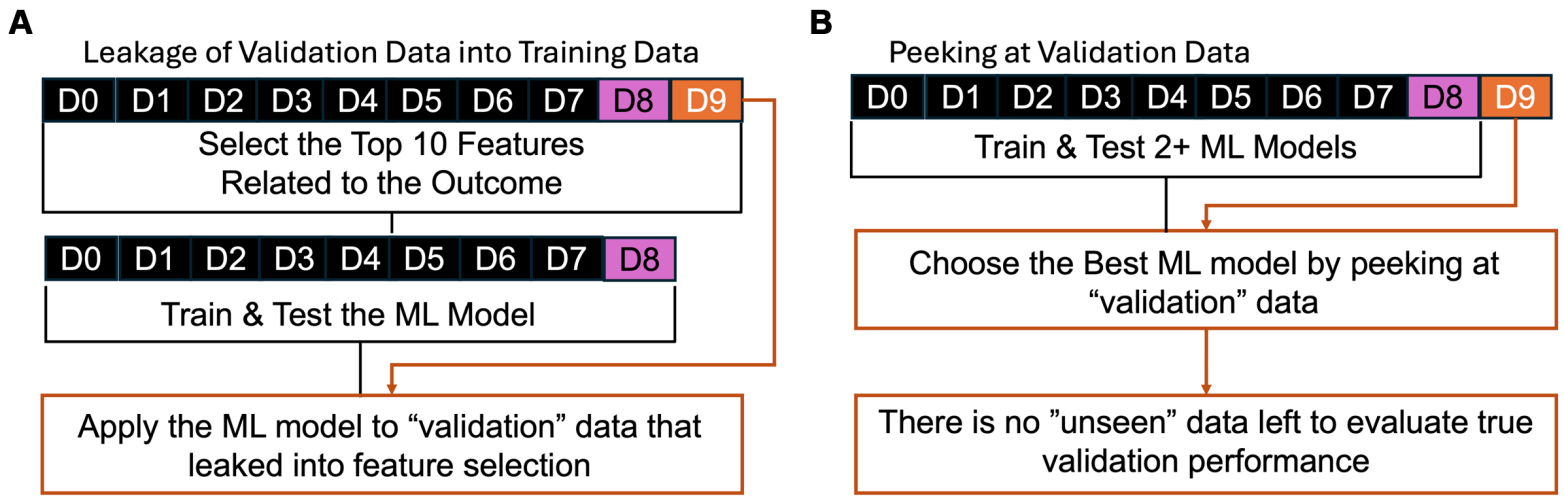
Examples of common errors of **(A)** “leakage” and **(B)** “peeking” where validation data is not truly “unseen.” In **(A)**, the validation data leaks into training by being used in feature selection to identify the features related to the outcome of interest. In **(B)**, the best performing ML model is chosen based on performance based on the “validation” data, but there is no data left to evaluate the performance of that best ML model on “unseen” data.

While we caution developers and clinicians interested in evaluating these technologies for “leakage” and “peeking,” there are good techniques to maximize the size of the training, testing, and validation datasets without acquiring three entirely independent datasets (48). In cross-validation, one dataset can be split into these components artificially according to assigned proportions (e.g., 80, 10, and 10%) or based on the number of people (e.g., leave-one-person-out). Additionally, in cyclic cross-validation, the assignment of data can cycle so that each piece of data is in the validation set once and only once (Figure 2). When there are no hyperparameters to learn and thereby no need for a “testing” set, there can be just one layer of cross-validation but when there are hyperparameters and a need for a “testing” dataset, one can perform nested cross-validation where the data are split into validation and a second dataset, then cross-validation is performed by splitting into training and testing within that second dataset to learn the hyperparameters (not pictured in Figure 2). The performance observed on the cycling validation data can then reflect the overall performance of the approach, even if the underlying ML/AI tool varies slightly from cross-validation fold to fold. This allows developers to develop high performance ML/AI tools based on large datasets, as well as validate them on data that was “unseen” by the tool. This approach of cross-validation and other similar techniques allow for development of new approaches on limited datasets, but before integration into clinical practice, these tools also should be validated with external datasets on a broad population of people (43–45).

**Figure 3:**
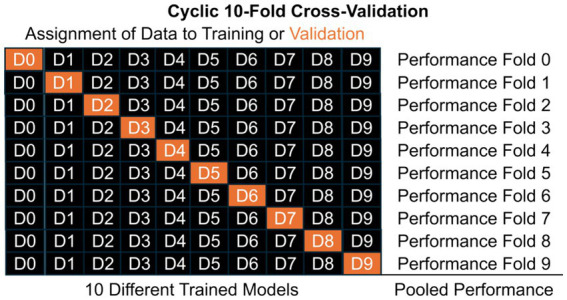
Illustrating the structure of a cyclic 10-fold cross-validation, where data is split into mutually exclusive subsets labelled D#. Model training occurs on training data only (black) and validation performance is estimated from validation data only (orange). In cyclic cross-validation, the identity of which data was validation cycles so that each subset of data is used for validation once and only once. Pooled performance across folds estimates performance of the general approach on unseen data, but each of the 10 different models likely have different learned parameters, *β*. When hyperparameters, *θ*, need to be learned, nested cross-validation can further split the black data into training and testing (pink in the Figure 1).

**Figure 4:**
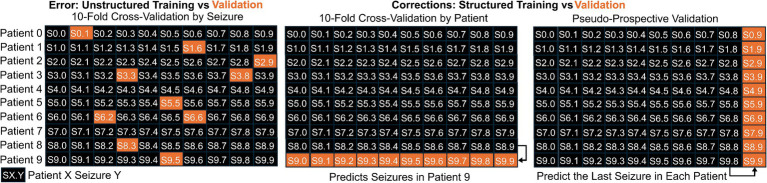
Illustration of the difference between splitting data into training and validation sets when the internal structure of the data is either maintained or modified. When the data includes 10 seizures from 10 patients, indicated by SX. Y for Seizure Y from Patient X, it would be an error to use unstructured splitting (first panel). Two appropriate methods for splitting into training and validation are illustrated. In the middle panel, we show training on data from 9 patients and validating based on the left out patient. In the right panel, we illustrate pseudo-prospective validation where the model is trained based on the first 9 seizures from each patient and validating using the last seizure from each patient.

**Figure 5:**
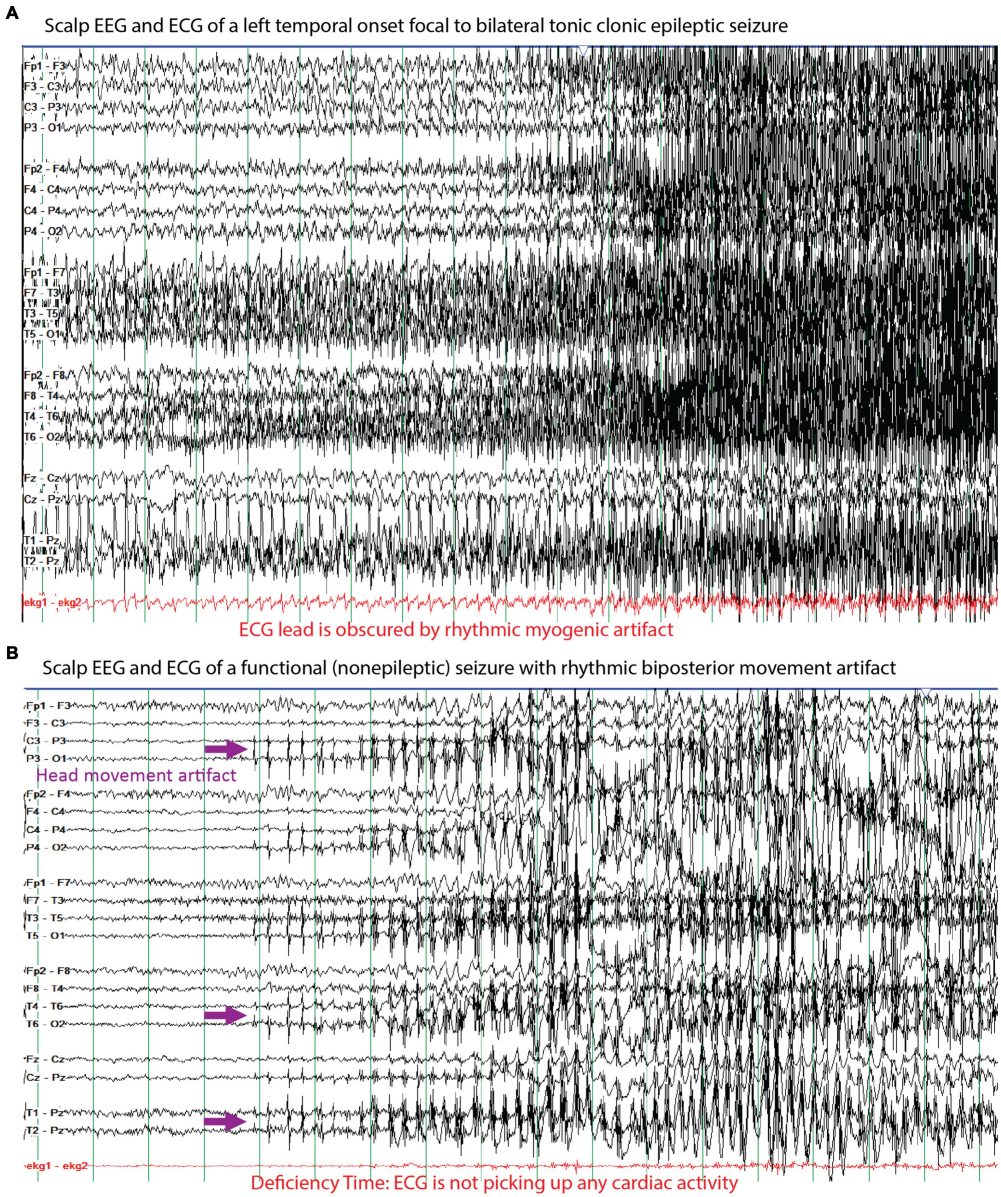
Examples of the electrographic, myogenic, and electrocardiographic (ECG) signals seen for **(A)** a left temporal onset focal to bilateral tonic clonic seizure and **(B)** a functional (nonepileptic) seizure with rhythmic artifacts. A challenge of seizure detection, prediction, and forecasting technologies are to differentiate these two types of events based on recording these signals with a combination of relevant sensors. The purple arrows highlight rhythmic artifact from side-to-side movement of the head against a pillow that appear to evolve like an electrographic seizure, but they can be differentiated from an epileptic seizure based on the high amplitude field in the posterior electrodes whereas the amplitudes in the anterior electrodes are markedly lower. The red markings highlight the challenges of ECG monitoring where in **(A)** the tonic-clonic movements include the chest and the muscle-generated signals obscure the relatively lower amplitude signals from the heart and in **(B)** we highlight that the ECG was not accurately recording during the seizure, which represents deficiency time.

**Figure 6:**
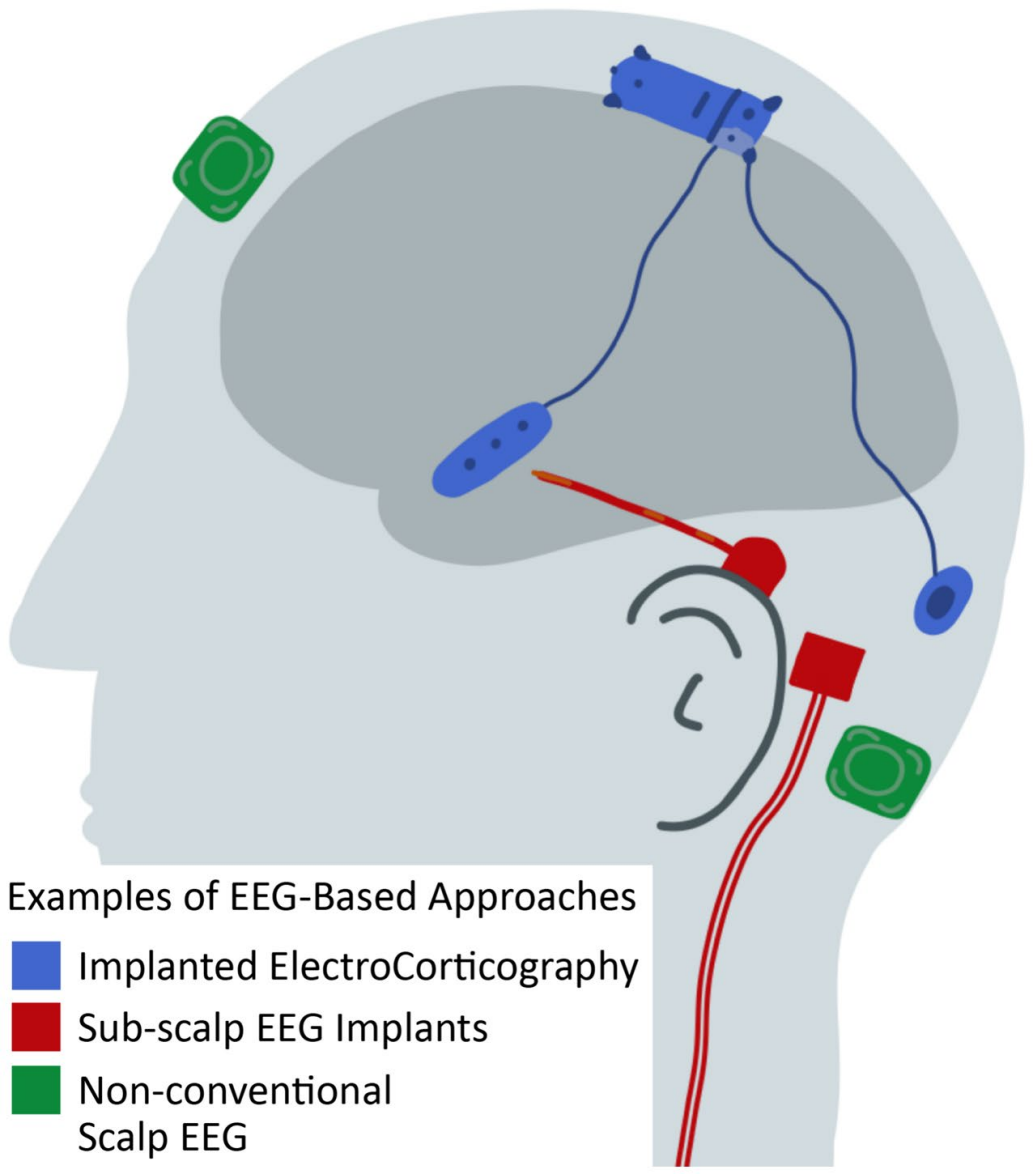
Illustration of EEG-based approaches for seizure detection, seizure prediction, and seizure forecasting that differ from conventional scalp EEG. See Table 1 and text for citations of specific technologies.

**Figure 7:**
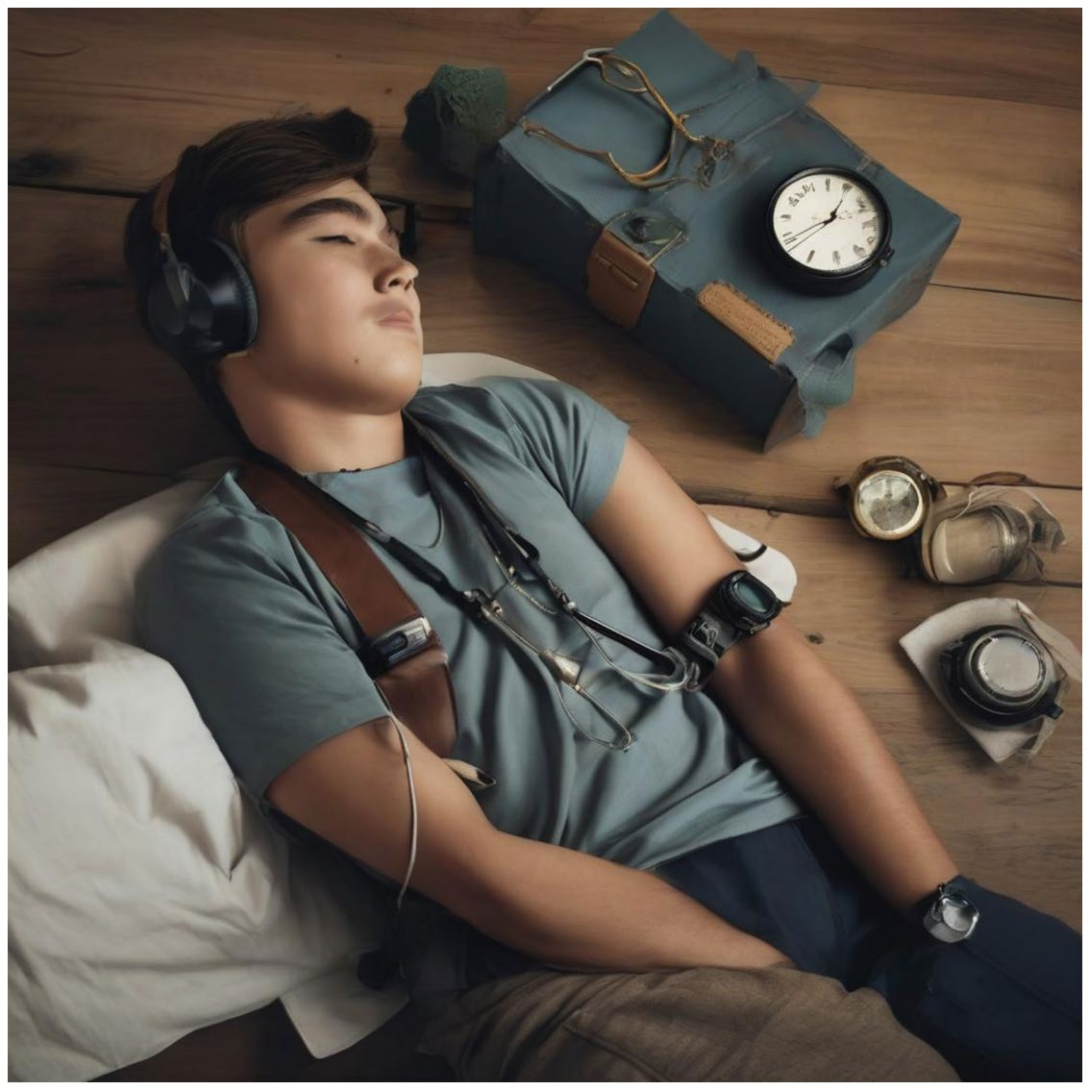
A GPT-4 generated illustration of a person wearing various monitoring devices that could be used for seizure detection, prediction, and forecasting. The white blanket could represent a bed pad monitoring device. The watch and bicep monitoring devices highlight where other external sensors can be placed. The headphones represent devices that can be worn around or inside the ear or head. See Table 2 and text for further descriptions.

The final common error that we will describe here is recognizing the internal structure in the data being from multiple people, each of whom may have had multiple seizures. Intuitively, data from the same person likely is more similar than data from different people, so if the data from one person serves within both training and validation, the models may have “seen” some aspect of the validation data. For example, using a nearest neighbor approach for seizure detection likely would identify the seizure most similar to the validation data, which probably would be from the same person! To account for this, developers can and should impose structure to the data splits of cross-validation where data are assigned to training, testing, and validation based on the person and not the individual time point. However, each person’s seizures often are stereotyped; therefore, performance can be improved substantially by understanding individual-level patterns, in addition to patterns common between multiple people. To accomplish that, training and test data for a real-time seizure detection or forecasting device could include only data from the same patient acquired before the validation data, which can be considered “pseudoprospective” validation (35, 58). This pseudoprospective validation occurs retrospectively, but by restricting to data available before the validation data, the goal is to simulate how the method would work when used prospectively (See Figures 3–7).

### 2.2 Understanding the metrics of performance

The traditional metrics to describe the performance of ML/AI tools describe how much the ML/AI tool predictions align with a gold-standard method. Outside of seizures, these metrics can include accuracy, sensitivity, specificity, predictive values, and receiver operating curves. These metrics often focus on data where the tools aim to differentiate between two conditions with relatively similar prevalence. This often is not the case for seizures. Ideally, continuous EEG monitoring for seizures in a patient who is critically ill observes zero seizures (59). When seizures are present, there often is fewer than 2 seizures, each lasting 2 to 5 min, within a 24 h recording. Therefore, even if a patient had 10 min of seizure in 24 h, any algorithm that naively predicted the patient was always not having a seizure based on classification of 1-min periods of EEG would have an accuracy of 99.3% (wrong for only 10 of 1,440 min). However, those 10 min are critically important to the care of that patient!

To overcome this, seizure detection, prediction, and forecasting tools typically focus on precision, recall, and calibration curves (60). In ML/AI, recall is equivalent to the sensitivity: the percent of positive cases (seizures) that were identified. Precision is equivalent to the positive predictive value: the percent of predicted positive cases (seizures) that indeed were positive cases based on the gold-standard metric. Analogous to a receiver operating curve that studies the relationship between sensitivity and specificity, there are precision-recall curves that display the relationship between precision and recall based on changing risk thresholds. The exact choice of risk threshold influences the calculation of precision and recall, therefore the area under the precision-recall curve; often abbreviated AUPRC, PR-AUC, or PRC; can summarize overall performance. This AUPRC is analogous to the area under the receiver operating curve for sensitivity and specificity.

In addition to predicting seizure versus not seizure, ML/AI tools often also provide a score or risk of seizure. The user can select the hyperparameter of risk threshold: scores above this threshold are called seizures and scores below this threshold are not seizures. Higher risk thresholds will have higher recall and specificity, but lower sensitivity and precision. One method to select these thresholds are by calibration curves, which display the relationship between the predicted risk and the observed risk with gold-standard diagnosis, which is equivalent to the precision of each risk threshold. One great example of a clinically relevant calibration curve is from the 2HELPS2B algorithm that predicts risk of seizure on continuous EEG in patients who are critically ill: a score of 0 indicates <5% risk of seizure after the first hour of monitoring, whereas a score of 1 or more indicates that 12 or more hours of seizure free EEG monitoring is needed for the risk of seizure on longer monitoring to be less than 5% (59, 61, 62).

In addition to these traditional metrics, seizure detection, prediction, and forecasting tools also must recognize the quality of their data by reporting a “deficiency time” (60). When sensors are worn for long periods of time, the quality of the signal often degrades so that it falls outside the realm of the training, testing, and validation data. This concept of degradation of quality over time is familiar for people who read inpatient long term conventional EEG, where affixing electrodes with tape allows for quality data for a couple of hours but collodion often is necessary for quality data for at least 24 h. Part of the engineering challenge for other technologies is to improve or maintain the quality of the signal, as well as the durability of that quality without technologist or human intervention. When the data is of insufficient quality to use the ML/AI tool, there is a distinct clinical difference between defaulting to a prediction of not seizure, as compared to identifying poor quality data. Therefore, these ML/AI tools should report a “deficiency time,” which is the duration of time where the data was of insufficient quality for detection, prediction, or forecasting (60). Unfortunately, because the datasets used to develop these ML/AI tools often are curated to be high quality, some developers neglect to evaluate deficiency time; therefore, users should be vigilant to consider this before utilizing the tool.

Each of these metrics of performance is estimated based on the validation data, which is a finite dataset. While it can be tempting to compare algorithms’ performance across datasets based on any one metric, it also is critical to recognize the uncertainty in each metric. For example, an accuracy of 80% in a sample of 100 patients is not statistically different from an accuracy of 82% in an independent sample of 100 patients (Fisher exact test Odds Ratio 0.88, 95% confidence interval 0.43–1.78, *p* = 0.86). Whenever comparisons are made between performances, readers should be diligent to make sure the comparison is based on rigorous statistical testing (56).

Often, summary metrics for machine learning are not normally distributed and often their distribution is unknown (e.g., variable importance). Common techniques to overcome these limitations are permutation testing and bootstrapping. In permutation testing, the link between the predictors (input data) and gold-standard outcome is broken by shuffling the rows of the outcome vector, without replacement. When the entire process of data cleaning, feature selection, training, testing, and validation are performed on a broken or permuted dataset, then the summary output should reflect chance performance. If this process is repeated for 10,000 independent permutations of the outcome vector, then one can build an empiric probability distribution for any summary metric. The traditional null hypothesis can be tested by asking if the observed metric (e.g., variable importance) was as extreme or more extreme than 5% of observed values on the permuted dataset (*p* < 0.05). A rule of thumb is that for this 5% threshold to be consistent when the permutation testing is repeated, at least 10,000 independent permutations should be done. For a threshold of 1%, then 50,000 independent permutations should be done. When algorithms are computationally intensive, these rules of thumb may not be practically possible. In comparison to permutation testing that empirically estimates the distribution of chance, bootstrapping takes the opposite tactic by estimating the distribution of observed performance. Bootstrapping creates datasets by randomly selecting data points (e.g., patients or seizures) with replacement so that, on average, some data points will be selected more than once. The entire process of machine learning can occur on these bootstrapped datasets to create an empiric probability distribution for the observed performance. While we advocate for permutation testing and bootstrapping, we also acknowledge that they are not appropriate in all cases (e.g., when the dataset is too small for 10,000 separate datasets to exist).

Additionally, the best comparison between ML/AI tools are made based on benchmark datasets (63, 64), where each algorithm is validated based on the same data. If this is not possible, then developers should ensure that the datasets used to measure performance are comparable: for example, it is not appropriate to compare performance of a seizure detection tool on critical care EEG to outpatient EEG (46, 47).

## 3 Seizure detection with EEG and electrocorticography

There have been decades of work by exceptional researchers on how to detect seizures using quantitative EEG features, but the problem remains unsolved (65–67). In this section, we describe the existing software and hardware approaches for seizure detection using traditional EEG, as well as long-term and wearable solutions (38).

The current standard for seizure detection using traditional clinical-quality EEG in critical care is SPaRCNet, which used a large dataset of long-term critical care scalp EEGs to train a deep neural network to identify electrographic seizures and other abnormal findings (46). To train this algorithm, there were nine independent board-certified epileptologists or neurophysiologists who annotated 15 s snippets of EEG. Unfortunately, one of the challenges for seizure detection is inter-rater agreement, which was an average of 55% for each pair of annotators. Therefore, SPaRCNet used the “gold-standard” of the majority vote of these annotators, which may or may not reflect a true “gold-standard” as compared to implanted intracranial direct ECoG in the appropriate region.

In addition to SPaRCNet, it is important to mention SCORE-AI, which similarly is an ML/AI tool used to interpret outpatient scalp EEG (47). While SCORE-AI also has impressive performance in the outpatient setting, the development dataset had a very limited number of epileptic seizures; therefore, its performance to detect seizures has yet to be determined reliably. Instead, it focuses on identifying interictal abnormalities.

Additionally, the intended application for ML/AI is to work in combination with human experts. To date, these technologies have been compared to human experts, but there has not been an evaluation of the performance of human experts with and without ML/AI assistance. Therefore, prior to wide adoption of these technologies, it should be shown directly that the human assisted by the ML/AI improves upon human performance without the ML/AI. We hypothesize that humans with ML/AI will be able to read EEG studies much faster with the same quality, but that study has not been done yet.

The most widely used and FDA-approved ML/AI tool for seizure detection using long-term ECoG is applied within the Responsive Neurostimulation System (RNS) (68–72). Patients with RNS have a small number of electrodes surgically implanted into the region thought to be the seizure onset zone or another area that can modulate seizures. Traditional programming of the RNS uses a small set of quantitative EEG features to identify when a patient is having an electrographic seizure and, in real-time, provide electrical stimulation to terminate the seizure. These programming settings are initially based on collaboration between an expert epileptologist and RNS engineer to maximize the sensitivity of prediction, provide stimulation as early as possible in the seizure, and minimize false positive rates to preserve battery life. In patients whose seizures do not improve in frequency or severity after neurostimulation programmed by human experts, there is an ML-based algorithm to suggest programming settings to optimize stimulation (73). These program settings are implemented using a human-in-the-loop approach where the ML-based algorithm suggests the programming settings, but human supervision is required prior to implementing those settings for the patient. This is our first example demonstrating that ML/AI tools do not currently aim to replace human experts, but the performance of human experts can be improved by collaboration with ML/AI tools.

In addition to these two more established technologies, there are numerous technologies in development that address the challenges of the established technology (Table 1) (38, 74, 75). The placement of the electrodes for the Responsive Neurostimulation System requires localization of the seizures, which can be challenging in some cases (73). In addition to other intracranial implants, there are technologies to implant targeted EEG recording electrodes underneath the skin or other tissues (76–80). After a small incision, these devices function for at least 1 month and, based on tolerability, have been used for at least 1 year. The next step down in invasiveness is targeted non-conventional scalp EEG systems, where the electrodes can be worn inside the ear or affixed to the skin through adhesives or placed within the ear to provide a limited coverage for days and perhaps up to a week or two (81–85). These non-conventional systems aim to replace ambulatory EEG systems, which often require an EEG technologist for placement and typically are limited to up to 72 h of monitoring (86).

**Table 1:** Categories of EEG-based approaches for seizure detection, seizure prediction, and seizure forecasting (illustrated in Figure 6).

EEG approaches	Target use case	Duration of use	Sensitivity	False positive rate	Key challenges
Implanted electrocorticography	Responsive neurostimulation	Lifelong	50–99%	1/month to 1/h*	Invasiveness of implant, implant location selection, clinical correlation, cost
Sub-scalp EEG implants	Monitoring and Forecasting	Months	40–99%	0.5/day to 4,768/h**	Clinical correlation, location selection, cost
Non-conventional scalp EEG solutions	Diagnosis and Characterization	Weeks	40–99%	0.5/day to 5/h	Skin reactions, sensor placement
Conventional scalp EEG	Critical and Emergency Care	Days	40–85%	0.5/day to 1/h	Skin reactions, EEG technologist time, cost

See text for citations of specific technologies. *Long-episodes within responsive neurostimulation can be proxies for seizures and sometimes detection and stimulation parameters are purposefully set to have a high false positive rate to allow for long-term neuromodulation. **Similarly, sub-scalp EEG can capture many electrographic seizures without a clear clinical correlate that can be unclear whether if they were true positive focal aware seizures versus false positives.

## 4 Seizure detection without EEG

One ultimate goal of seizure detection technologies is to produce automated and reliable counts and diagnoses of epileptic seizures based on long-term wearable devices (Table 2) (87–90). If a person with seizures could wear a device with high sensitivity for seizure detection and low false positive rate, then the device could alert caregivers, emergency services, and others to when the person with seizures requires assistance which, in turn, could have a direct impact upon risks of Sudden Unexpected Death in Epilepsy (SUDEP), early treatment of status epilepticus, and monitoring for treatment response (90). The challenges to seizure detection without EEG is that the device must (1) capture data relevant to the seizure, (2) reliably differentiate seizure from non-seizure, and (3) be wearable.

**Table 2:** Categories of non-EEG based approaches for seizure detection, seizure prediction, and seizure forecasting (illustrated in Figure 7).

Non-EEG approaches	Target use case	Duration of use	Sensitivity	False positive rate	Key challenges
Bed mattress	Nocturnal convulsive seizures and SUDEP	Months to Years	62–89%	3/year to 0.5/night	Nonconvulsive seizures, sleep behaviors, sensor placement
Arm-worn (biceps)	Convulsive seizures	Days to Months	75–90%	0.5/day to 6/night	Wearability, correct sensor placement, movement artifacts
Wristwatch	Convulsive seizures	Days to Months	80–95%	0.25/day to 1.2/day	Wearability, movement artifacts, noisy data
In Ear*	Convulsive and Electrographic seizures	Hours to Days	55–99%	5–60% of all detections	Wearability for long term use, restricted to near-ear epilepsy

See text for citations of specific technologies. The in-ear technology can use EEG in addition to non-EEG signals for seizure detection.

Current clinical decisions are made based on quasi-objective seizure diaries where patients and their caregivers keep track of a rough seizure count. Based on this rough seizure count, clinicians attempt to judge whether seizures change in response to modifications in treatment. Analysis of the electrocorticography from the implanted Responsive Neurostimulation System indicated that treatment response could be predicted within a week of reaching the equilibrium dose of an ASM, but that requires intracranial surgery (25). If similar quality of seizure counts could be achieved with less invasive monitoring, then treatment responses could be made much more quickly and reliably.

There are many non-electrophysiological methods to monitor for seizures including accelerometry (ACM), electromyographic (EMG), cardiac monitoring, electrodermal activity (EDA) to measure sweating, and photoplethysmography (PPG) to measure blood oxygenation with light (90, 91). As discussed in Section 2, these technologies are primarily judged based on their sensitivity (what percent of seizures do they capture), false positive rate (what percent of alerts are indeed seizures), and deficiency time (what percent of the time they capture quality data) (60).

The principle behind accelerometry is that many seizures either produce movements or represent an absence of movement or tone. With objective recording with a wearable device, the detailed characteristics of these movements have been able to differentiate bilateral or generalized tonic-clonic epileptic seizures from mimics including functional seizures (otherwise known as psychogenic nonepileptic seizures) as well as other transient neurological conditions like convulsive syncope (92–94). This has been demonstrated in small to moderate size studies, but the performance of these technologies has not been demonstrated in the broad and medically complex populations typically seen in outpatient clinic or even video-EEG monitoring units. The first evaluation of these devices typically is in patients admitted in a highly controlled environment for video-EEG monitoring to directly compare the device to the gold standard. Once evaluated there, devices can then be utilized in the real world. These subsequent evaluations have shown that many behaviors like clapping, walking, running, and other repetitive motor behaviors can be challenging to differentiate from seizures. In some patients, this can produce as many as two false positive seizure detections a day, which would be intolerable in the context of having one epileptic seizure every 3 months (95, 96).

In addition to accelerometry, wearable sensors can evaluate other aspects of a person’s health (90). Smart watches are increasingly prevalent and can directly associate seizures with cardiac cycling. In fact, there is emerging work demonstrating synchronicity of both circadian and multi-day cycles of seizures and heart rate variability (97). Due to the inherent limitations of accelerometry, the addition of multimodal sensors has the great potential to improve upon both sensitivity and false positive rate. The addition of sensors also may increase the invasiveness of the technology and thereby create wearability challenges if the patient can only tolerate wearing the sensors for a restricted period. For example, bed covers are a good example of being very wearable because they only change the feel of the bed, but also miss seizures that do not occur in bed. In contrast, the Responsive Neurostimulation System (RNS) requires surgical implantation, daily to weekly data uploads, and in person clinic visits roughly every 3 months for programming. Other technologies balance between these two extremes of least invasive to most invasive. Table 2 summarizes some of the current approaches using multimodal sensors for seizure detection.

## 5 Seizure prediction and forecasting

While seizure detection itself is valuable because it can improve patients’, caregivers’, and the healthcare system’s responses to seizures, it would be even more powerful if we could predict when seizures would occur in the future (33, 34, 98). The practical definition of epilepsy is based upon a greater than 60% 10 year cumulative risk of unprovoked seizures (99), but that the risk of seizures is not the same on each individual hour, day, week, or year. Some of the key aspects of the disability incurred by recurrent seizures is the lack of predictability. A fear of seizure can contribute to a new onset or worsening anxiety disorder (32, 37). Similarly, the feeling of learned helplessness from being unable to control when seizures occur can contribute to the high rate of comorbid depression in people with seizures (100). If individual minutes or days with greater than 60% risk of seizures could be predicted reliably, then practical actions could be taken to improve both quality of life and safety, like taking additional medication(s), alerting a caregiver, or not driving an automobile. In days of low seizure risk (e.g., <1%), a radical suggestion would be that people with seizures could be indistinguishable from people without seizures and may not even require ASMs, similar to oligoepilepsy (101). Unfortunately, our methods for seizure prediction and forecasting are not yet good enough to achieve that vision for the future (102).

In addition to producing practically useful tools, the process of developing seizure prediction and forecasting methods improved our understanding of the continuum of states from inter-ictal, pre-ictal, ictal, to post-ictal. These states differ from the reporting guidelines for expert interpretation of EEG, which focus on differentiating inter-ictal activity from ictal-interictal continuum and electrographic or electroclinical seizure. In some of the pivotal work on seizure prediction, intracranial monitoring with NeuroVista demonstrated that in order to have a seizure, many patients first transition to a pre-ictal state where the likelihood of seizure is high, but seizure is not guaranteed (103, 104). In the inter-ictal state, the risk of transitioning directly to seizure within seconds or minutes is very low (e.g., 1/year), but the risk of subsequent seizure in the pre-ictal state is higher (e.g., 1/week). This is consistent with other observations about the propagation of the epileptic activity from the seizure onset zone to a symptomatogenic zone that produces clinical symptoms including, but not limited to, “auras” or focal aware seizures in isolation (105, 106). Many patients with focal-onset seizures have focal aware seizures that do not progress to focal unaware seizures. In contrast to focal aware seizures (auras), these pre-ictal states are not seizures. However, recognition of pre-ictal states could represent an opportunity for intervention to avoid progression to seizures (See Table 3).

**Table 3:** Glossary of common and important terms in the field of machine learning and artificial intelligence for seizure detection, prediction, and forecasting.

Term	Abbreviation	Definition
Artificial intelligence	AI	Tools trained using historical data to perform a broad array of tasks mirroring human intelligence, including tasks for which they have not been explicitly trained
Machine learning	ML	Algorithms trained using historical data to maximize performance on a specific task
Deep learning	DL	ML algorithms that use multiple, “deep,” layers of data processing to improve performance
Support vector machine	SVM	A type of machine learning algorithm that identifies a maximum separating hyperplane between training data based on the hardest to classify examples, called “support vectors”
Neural networks	NN	A type of machine learning algorithm that commonly uses multiple layers of hidden combinations of the input data, called nodes, to identify complex patterns in the data that may improve performance. The interconnections of these hidden nodes often are modeled based on the connections of neurons. This technique is commonly used in DL
Training set	–	Historical data that ML/AI algorithms use to learn patterns in data
Testing set	–	Data separated from the training set that is used to train higher level structures of ML/AI algorithms (e.g., which ML/AI algorithm is superior to which)
Validation set	–	Data separated from the training and test sets that is used to estimate performance when applied to unseen data
Feature	–	Quantitative data that can be input into ML/AI algorithms to perform predictions. Also known as independent variables or predictors
Feature selection	–	Identifying a subset of the input data that is most related to the outcome of interest
Peeking	–	An error in ML/AI tool development where “validation” data is used in training or testing (e.g., choosing the superior ML/AI method on a dataset)
Leakage	–	An error in ML/AI tool development where “validation” data leaks into some stages of training or testing (e.g., feature selection)
Bootstrapping	–	Empiric estimation of the variability of results by repeating the analyses on datasets where data was randomly selected with replacement
Permutation testing	–	Empiric estimation of the variability of chance or null hypothesis results by repeating the analyses on datasets where the outcome of interest is randomly shuffled without replacement
Sensitivity/Recall	–	The percent of positive outcomes (e.g., seizures) that was accurately identified
Positive predictive Value/Precision	PPV	The percent of outcomes predicted to be positive that indeed were positive (e.g., seizures)
False positive rate	FPR	The rate that the ML/AI algorithm predicts a seizure occurred when a seizure did not occur
Deficiency time		The percent of time when the device or ML/AI algorithm is not recording high enough quality information to make a reliable prediction of outcomes
Area under the receiver operating curve	AUC	Area under the receiver operating curve of the balance between sensitivity and specificity
Area under the PR-curve	PR-AUC PRC	Area under the curve showing the balance between precision and recall

If this pre-ictal state can be identified reliably, then targeted changes to treatments can be implemented to transition back to the inter-ictal state as compared to the ictal state (33). For example, Chiang and colleagues demonstrated that the different types of neurostimulation were effective to reduce seizures when applied in a pre-ictal state as compared to an inter-ictal state (107). Even when seizures do not occur in someone who is seizure free, identification of these transitions to a pre-ictal state could delineate the risk of seizure with medication withdrawal. Recruiting for randomized trials for medication withdrawal can be challenging because if someone is seizure and side effect free on antiseizure medication(s), then there may not be motivation to stop the antiseizure medication(s). Lowering or stopping antiseizure medications in this context can reduce healthcare costs and adverse effect burden, but there’s always a risk of breakthrough seizures when coming off medications and even one breakthrough seizure can have big implications (e.g., driving restriction, injuries from seizures, and SUDEP). In addition, it can be challenging to train a machine learning algorithm to identify seizures in patients who are seizure free because there may be no examples of an individual’s seizures captured with the device for training (see Section 2 for discussion of internal structures in data). While training based on others’ seizures can somewhat translate to individual patients, these seizure prediction and forecasting methods often require personalized modifications (26). Especially because there is no electrographic definition of a pre-ictal state, it is not yet possible to reliably identify this preceding state without observing the seizure that it precedes.

These concepts of state transitions have led the field to transition from seizure prediction to seizure forecasting (34). In seizure prediction, technologies aim to make clear yes or no predictions that a seizure will or will not occur. In seizure forecasting, the purpose is to provide a risk-gauge for seizures that likely differs from these clear yes and no insights. Like weather forecasting, these seizure forecasts are most helpful when the risk substantially varies with time so that different decisions can be made based on this estimated risk, but also recognizing that the predictions are imperfect.

One of the insights from seizure forecasting is that continuous technological monitoring may not be necessary to provide reasonable seizure forecasts (98). Part of the phenomenology of Juvenile Myoclonic Epilepsy is the circadian cycle where myoclonic jerks and seizures are more common in the early morning. Those circadian cycles also are present in seizures observed in continuous video-EEG monitoring units and critical care (40, 103, 104, 108, 109). However, these circadian cycles may not be as prominent as suggested by the data, because there may be a confound of circadian under-reporting of seizures (e.g., nocturnal focal aware seizures are missed because patients are sleeping, causing the data to suggest that focal aware seizures only occur during the day) (110).

In addition to circadian rhythms, the multi-day cycles in seizures have been well recognized (103, 108, 111). Catamenial or menstrual cycle related seizure patterns have long been recognized based on patient and caregiver observations, with subsequent work demonstrating sub-patterns of catamenial seizures based on the relative sensitivity to the pro-convulsant effect of estrogen and the anti-convulsant effect of progesterone (112, 113). Recognition of these patterns can prompt cyclic prescription of antiseizure medications, where a higher dose is taken 3 days before, during, and 3 days after the “high risk” period (113).

The addition of long-term wearable technologies has enhanced our recognition of these seizure cycles being present in as high as 30% of people with medication resistant epilepsy (111, 114). When seizure diaries were paired with long-term cardiac monitoring, Cook and colleagues recognized that the monthly cycles of seizures occur in men with a similar prevalence as catamenial epilepsy (97). They also observed synchrony of the heart rate variability in concurrent cardiac monitoring with a watch, as compared to seizure cycles, which prompted hypotheses about the mechanisms of SUDEP (2). In addition, there commonly are empiric cycles with differing lengths, including weekly seizures, fortnightly seizures, and even multi-month patterns (111, 114). When these patterns are identified in a person, then additional clinical questions arise regarding potential cyclic non-adherence to medications (e.g., seizures on Mondays after missing weekend antiseizure medication doses) or other associations with external or internal factors (e.g., sleep deprivation, head injuries, hormonal changes). Other multi-day fluctuations may be based on the body’s intrinsic and adaptive resilience to seizures through balancing excitation and inhibition within epileptic networks.

An important concept to identifying these seizure patterns is the Nyquist frequency, which some providers may be familiar with based on interpreting EEG. The Nyquist frequency states that to observe a signal with a certain frequency, then the sampling rate must be at least twice as fast or twice as long as the target frequency. In EEG, this means that frequencies faster than 125 Hz cannot be observed if the EEG data has a sampling frequency of 250 Hz. For seizure cycles, to measure a monthly seizure cycle, there must be at least 2 months of data. Based on this concept, devices that use monthly cycles for seizure prediction and forecasting must be worn for at least 2 months before there is sufficient data from the individual patient to measure that cycling. This represents a technological minimum and the reliability of measuring these cycles can improve if longer monitoring is performed. To highlight that the Nyquist frequency is the technological minimum, the International League Against Epilepsy uses other statistical principles to define a response to treatment by an at least tripling of the between-seizure interval, which can be called the “Rule of Three.” (99).

The above concepts of seizure prediction and forecasting primarily are within the realm of research and have not been demonstrated as directly clinically applicable yet (102). When thinking about the future of machine learning and artificial intelligence, we recognize that the current existing tools are the worst that we will see in the future (43). The goal of researchers, engineers, and clinicians is to use our constantly improving capabilities for data processing and understanding to improve upon these standards. While healthcare is commonly very slow to migrate, we recognize the great capacity for machine learning and artificial intelligence to make massive disruptive changes. For example, the public release of large language models like ChatGPT have forever revolutionized the practice of essay writing in higher education (115, 116). Even though these disruptive technologies do not exist for seizure detection, prediction, and forecasting yet, we must begin to understand both their potential benefits and limits so that when, not if, these technologies exist, then we are able to utilize them responsibly (43).

## 6 Implications for clinical trials

One initial use for these technologies for the detection, prediction, and forecasting of seizures is in clinical trials of treatments for epilepsy. Clinical trials are the foundation upon which we determine the efficacy of novel treatments, but there are increasing challenges to the design and conduct of trials (14). These challenges are highlighted by the reducing number of participants per site recruited, which requires a compensatory increase in the number of sites to meet sample size goals (14).

However, this increase in sites also has been associated with a progressive increase in the placebo response rate, which likely harms the statistical power of trials by shrinking the difference between the elevated placebo and the efficacious treatment, which may be capped by a ceiling effect (14, 16). That creates a vicious cycle where higher placebo response rate reduces statistical power, which increases the number of participants and sites which, in turn, further increases the placebo response rate.

The insights that we have gained from seizure detection, prediction, and forecasting work have both highlighted those problems as well as offered potential solutions. The challenge in recruiting participants in a site is that more than 50% of patients with medication resistant epilepsy seen at a tertiary care center for epilepsy with a long history of participating in clinical trials did not meet the minimum seizure frequency requirement (117). To be able to observe the efficacy of treatment within a reasonable time frame of a 12 or 20 week maintenance period, focal-and generalized-onset seizure trials often require at least 4 seizures per month and 3 seizures per 2 months, respectively. Lowering those minimum seizure frequency eligibility requirements may necessitate longer maintenance periods, but longer maintenance periods further increase cost and hinder participant recruitment because placebo exposure in blinded trials was associated with a 5.8-fold increased risk of SUDEP (118).

One of the challenges incurred by these minimum seizure frequency requirements called regression to the mean can be understood using the language of seizure forecasting (119). In regression to the mean, a participant is motivated to enroll in a placebo-controlled trial of a new antiseizure medication due to a progressive recent increase in seizure frequency from 1 per month to 4 per month for 3 months (119). Now that they are eligible to participate, they start keeping a prospective seizure diary during the 4-to-8-week pre-randomization baseline. If this recent increase in seizure frequency was due to inherent changes in their seizure cycles causing an increase in their rolling-average seizure forecast of 4/month, then they may spontaneously transition back to that low seizure frequency state (1/month) during the baseline and be a “baseline failure.” However, if this spontaneous transition occurs after the pre-randomization baseline and instead during the blinded randomization phase, then their 75% reduction in seizure frequency from 4/month to 1/month may be unrelated to the efficacy of their blinded treatment. While trials do not report the reason for “baseline failures,” the increase in placebo responder rate has been associated with an increasing rate of baseline failures (14).

In addition to giving us the language to describe those changes, seizure forecasting can provide solutions. The seizure cycling literature demonstrated that, by and large, most seizure cycles are less than 1 month long. Therefore, the minimum duration to evaluate the efficacy of a treatment likely is longer than 1 month. Trial design can use this knowledge to create adaptive designs, where participants engage in blinded treatment until there is sufficient data to conclude that they have either responded or not responded to the blinded treatment, irrespective of the length of observation. One proposal to accomplish this is through the Time to Prerandomization monthly Seizure Count (T-PSC) design, where participants engage in blinded treatment until their blinded post-randomization seizure count exceeds their average monthly seizure count measured during baseline (13, 120). This design allows for quick exclusion of participants who worsen on blinded treatment (either due to nocebo or worsening of seizures on novel treatment), who likely have an increased risk of SUDEP and other serious adverse events (118). [The nocebo effect is defined as a negative effect of placebo treatment, including either worsening of seizures or adverse effects (20)]. Simultaneously, this design also either maintains the traditional 12-to-20-week endpoint for initial responders. This traditional 12-to-20-week endpoint remains necessary to differentiate novel treatments from short-acting benzodiazepines, which transiently lower seizure frequency but do not have a sustained effect. Re-analyses of numerous historical trials has demonstrated that the efficacy conclusions of the trials truncated at T-PSC were almost identical to the conclusions with full-length trial data (13).

The other challenge of clinical trials is producing reliable and accurate counts of epileptic seizures (11). Foundationally, the approval of novel treatments is based upon demonstrating both the magnitude of the difference in efficacy between placebo and active treatment. While patient-and care partner-reported diaries are the current standard for clinical trials, there is substantial data suggesting that the sensitivity of these reported seizure detections is less than 50%, where some seizure types may have even lower sensitivity (e.g., focal aware seizures, nonmotor focal unaware seizures, absence seizures) (24). There also may be circadian patterns in reduced sensitivity that may create artificial circadian patterns in seizure diary data (e.g., reduced sensitivity during sleep) (110). Theoretically, this reduced sensitivity increases the variability of seizure count by adding a source of noise, as well as lowering the apparent seizure frequency in each phase of the trial, which can impact the certainty in measuring each seizure frequency as well as eligibility (e.g., insufficient baseline seizures to progress to the blinded phase).

In addition to increasing the variability of the difference estimate within trials, imperfect human-provided seizure counts can incur bias (110). While bilateral or generalized tonic-clonic seizures likely have low false positive rate of reporting, data from ambulatory and inpatient EEG monitoring suggest that patients and care partners also may have an unclear false positive rate due to inaccurate differentiation of other transient neurological symptoms from their seizures (121). Especially for scalp EEG negative focal aware seizures, it can be very challenging to evaluate seizure from non-seizure events. Theoretically, false positive seizure counts cause under-estimation of difference between placebo and active treatment because true seizures may benefit from treatment, but false positive counts would not.

These limitations in human-provided seizure diaries clearly could be assisted by objective devices that improve upon both the sensitivity and false positive rates. Long-term wearable or implanted devices could provide more accurate time stamps on seizures and provide further characterization of many details regarding seizures (e.g., duration of the motor phase) that often is too burdensome for humans to reliably record. Combined with the development of seizure prediction and forecasting methods, these granular data can incorporate the known confounding factors of seizure cycling and clustering into improved quantitative metrics measuring improvements in seizures.

To achieve these future benefits, many devices have been shown to improve the sensitivity of seizure detection but, unfortunately, these devices also incur a false positive rate that may be higher than humans (90). Additionally, some of the long term intracranial, subcutaneous, or other EEG-based devices may reliably measure electrographic seizures, but there is an incomplete link between electrographic and electroclinical seizures (74). Improvement in electrographic seizures likely does not guarantee an improvement in electroclinical seizures, the latter of which likely have a greater impact upon quality of life. These devices also often have demonstrated this performance in selected patients who are interested in enrolling in studies of the devices, as compared to a broader population who would enroll in a trial for a novel treatment.

Each of these concerns can be addressed through further study and validation of existing devices, as well as improving upon existing technology. Lastly, clinical trials are both very expensive and high risk for sponsors who often seek to mitigate that risk through following conventional trial protocols. While integrating devices into trials could have some benefits, providing innovative devices to trial participants would increase cost, as well as risks. When (not if) a device for seizure monitoring is used in a trial, there is a nontrivial risk that the human-provided seizure outcome may differ from the device-based diary. (To our knowledge, such a trial has not been completed and published yet but, to our knowledge, there indeed is a trial underway that combines both human diaries and long-term EEG based seizure detections). To describe this risk, consider the options for when the human and device-based diaries disagree. If the human-provided diary suggests benefit but the device does not, then clinicians, sponsors, and regulators may conclude that the treatment benefitted clinically meaningful seizures because humans counted them. However, the trial would conclude that the treatment does not have an impact on the device-based seizure counts, which may be felt to not be clinically meaningful. Conversely, if only the device demonstrated treatment benefit, then the conclusion would be that this likely would be a statistically significant, but not clinically significant, improvement. This risk of discrepancy could be mitigated by using a human-in-the-loop system where the device’s seizure predictions are provided to the patient and care partner for supervision, as well as subsequent review by study staff or treating clinicians (122). However, these human-in-the-loop studies have not been done, to date, within epilepsy or seizures.

As researchers with expertise both in machine learning and clinical trials, we believe that clinical trials will eventually rely upon seizure counts assisted by long-term seizure detection and monitoring devices that use innovative machine learning techniques to improve upon the current standard of human-provided seizure diaries. Each of the concerns that we have raised and others that we have heard are addressable through further development of both the hardware and software for seizure detection, prediction, and forecasting.

## 7 Conclusion

ML and AI will revolutionize the clinical care of people with seizures, but the field is nascent. In the future, both software and hardware will improve the reliability of the original diagnosis of epilepsy compared to mimics, monitoring of seizure recurrence with treatment, evaluating novel treatments within clinical trials, and providing a real-time and actionable warning system that people with seizures can use to improve safety and quality of life. While these improvements are promising, the performance of the existing technology has not been demonstrated to be high enough to warrant a change in current clinical practices. We are confident that in the near future ML and AI will not replace clinicians, but clinicians assisted by ML and AI will replace clinicians not utilizing ML and AI.

## Author contributions

WK: Conceptualization, Funding acquisition, Writing – original draft, Writing – review & editing. KM: Conceptualization, Writing – review & editing. GF: Conceptualization, Visualization, Writing – review & editing.
